# Supplementary material for: Cis- and trans-resveratrol have opposite effects on histone serine-ADP-ribosylation and tyrosine induced neurodegeneration
Source: Nat Commun. 2022 Jun 10;13:3244. doi: 10.1038/s41467-022-30785-8 (PMC9187644; doi:10.1038/s41467-022-30785-8)
Supplement: Supplementary file 1 — Supplementary Information [file 41467_2022_30785_MOESM1_ESM.pdf]

Supplementary Information

Cis- and *trans*-Resveratrol Have Opposite Effects on Histone Serine-ADP-Ribosylation and Tyrosine Induced Neurodegeneration

Megha Jhanji[1], Chintada Nageswara Rao[1], Jacob C. Massey[1], Marion C. Hope III[1], Xueyan Zhou[2], C. Dirk Keene[3], Tao Ma[2], Michael D. Wyatt[1], Jason A. Stewart[4], and Mathew Sajish[1*]

[1]Department of Drug Discovery and Biomedical Sciences, College of Pharmacy, University of South Carolina, SC 29208
[2]Department of Internal Medicine, Gerontology and Geriatric Medicine, Wake Forest School of Medicine, Winston-Salem, North Carolina, USA
[3]Department of Laboratory Medicine and Pathology, University of Washington School of Medicine, Seattle, Washington, USA
[4]Department of Biological Sciences, College of Arts and Sciences, University of South Carolina, SC 29208

*Correspondence: mathew2@cop.sc.edu

Supplementary Figure 1

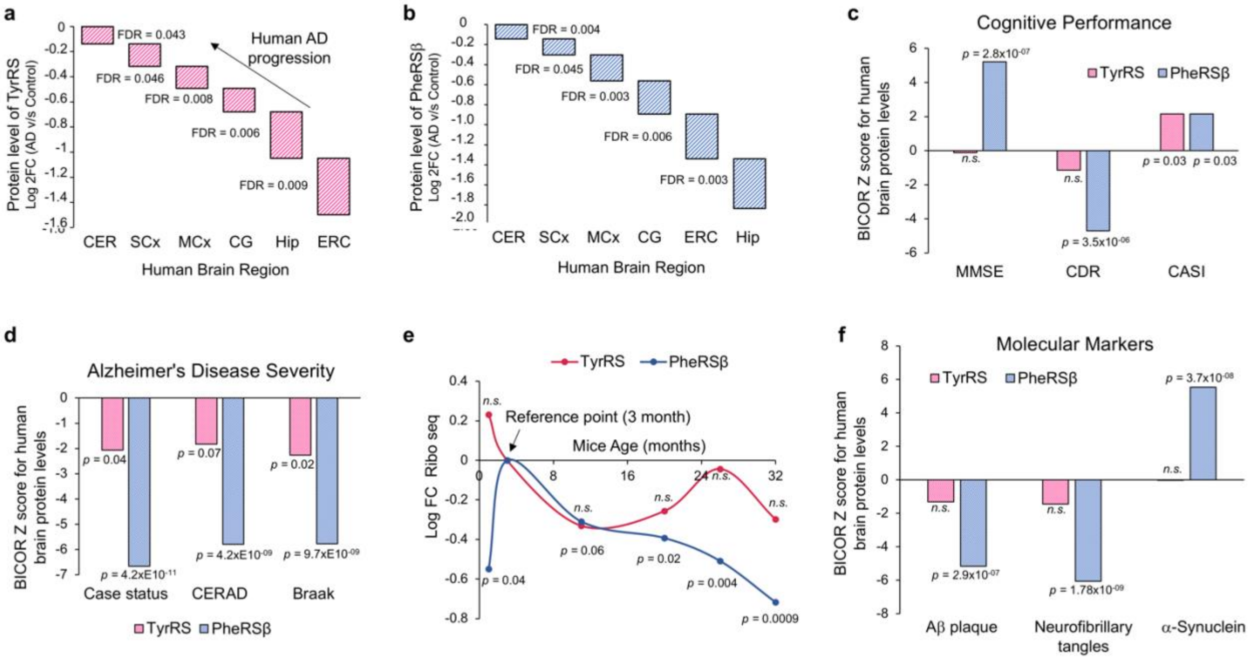

Supplementary Figure 1. Human brain protein levels of TyrRS and PheRSβ correlate with cognitive performance and AD severity, but TyrRS levels do not correlate with known molecular markers of neurodegeneration. a, b. *TyrRS (**a**) and PheRSβ (**b**) are decreased in disease-affected brain regions of AD patients.* Graph created by the re-analysis of published data from Xu *et al.* (2019)[1]. Changes in protein levels of TyrRS from six functionally distinct regions of human post-mortem brain of AD cases versus asymptomatic controls: entorhinal cortex (ERC), hippocampus (Hip), cingulate gyrus (CG), sensory cortex (SCx), motor cortex (MCx) and cerebellum (CER) identified using mass spectrometry. Donors (n = 9 AD cases, n = 9 asymptomatic controls) were well matched for age and post-mortem delay. Proteins with an average local false discovery rate (FDR) ≤ 5% were deemed significant. Data represented in box plots using average, minimum and maximum values. **c** and **d.** *Brain protein levels of TyrRS and PheRSβ correlate with human cognitive performance and dementia*. Biweight midcorrelation (BICOR) Z scores of TyrRS and PheRSβ were created using data published by Johnson *et al.* (2020)[2]. Increased brain TyrRS and PheRSβ levels show a positive correlation with human cognitive function (**c**), and its decrease correlates with AD case status and disease severity (**d**). **e.** *Mouse aging correlates with decreased ribosome occupancy of mRNA of PheRSβ, not TyrRS.* Graph created by the re-analysis of published data from Anisimova, A. S. *et al.* (2020)[3]. Mice livers representing six age groups (1-, 3-, 11-, 20-, 26-, and 32-month old) were used. The dynamics of age-related changes in ribosomal coverage of mRNAs were assessed by obtaining a list of nonoverlapping segments with stable footprint coverage and normalized to the mean coverage at three month. **f.** *Brain TyrRS levels do not correlate with known neurodegenerative disease markers*. BICOR coefficients of only PheRSβ, not TyrRS shows a significant correlation with known molecular markers of neurodegenerative diseases. Data represents Z scores and p values for protein correlations.

Supplementary Figure 2

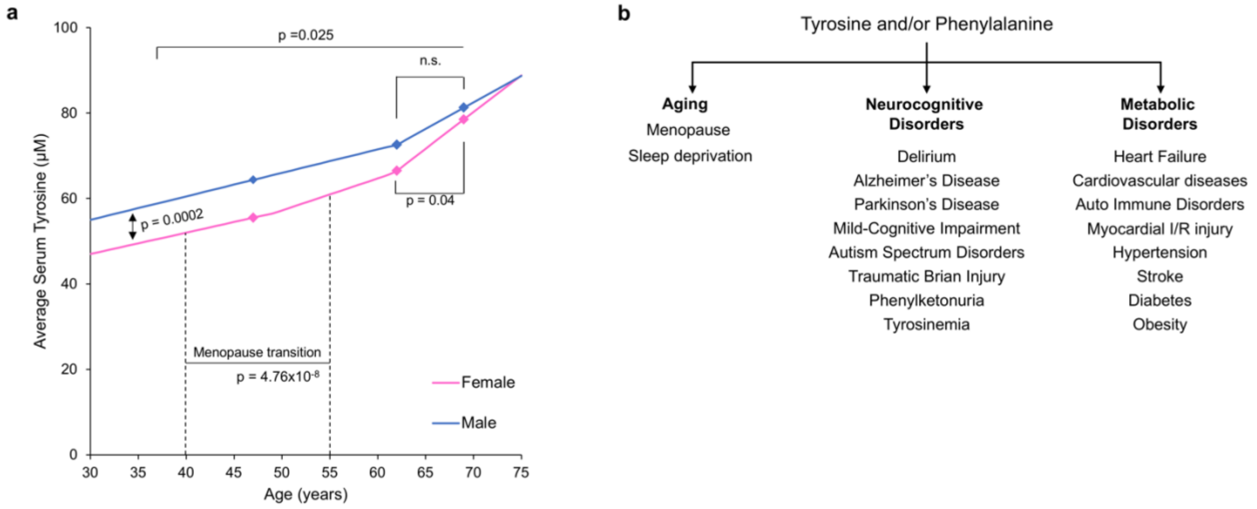

Supplementary Figure 2. Tyrosine is lower in young women but increased during human aging, and in patients with neurocognitive and metabolic disorders. a. *A model of age-dependent increase in serum tyrosine concentrations.* The model graph was built using data obtained from different longitudinal metabolomic studies assessing the effect of aging on metabolic profiles along with their study demographics mentioned in Supplementary Table 2. **b**. Illustration of various neurocognitive and metabolic disorders with reported increased in serum Tyr and/or Phe as reported in works mentioned in Supplementary Table 3.

Supplementary Figure 3

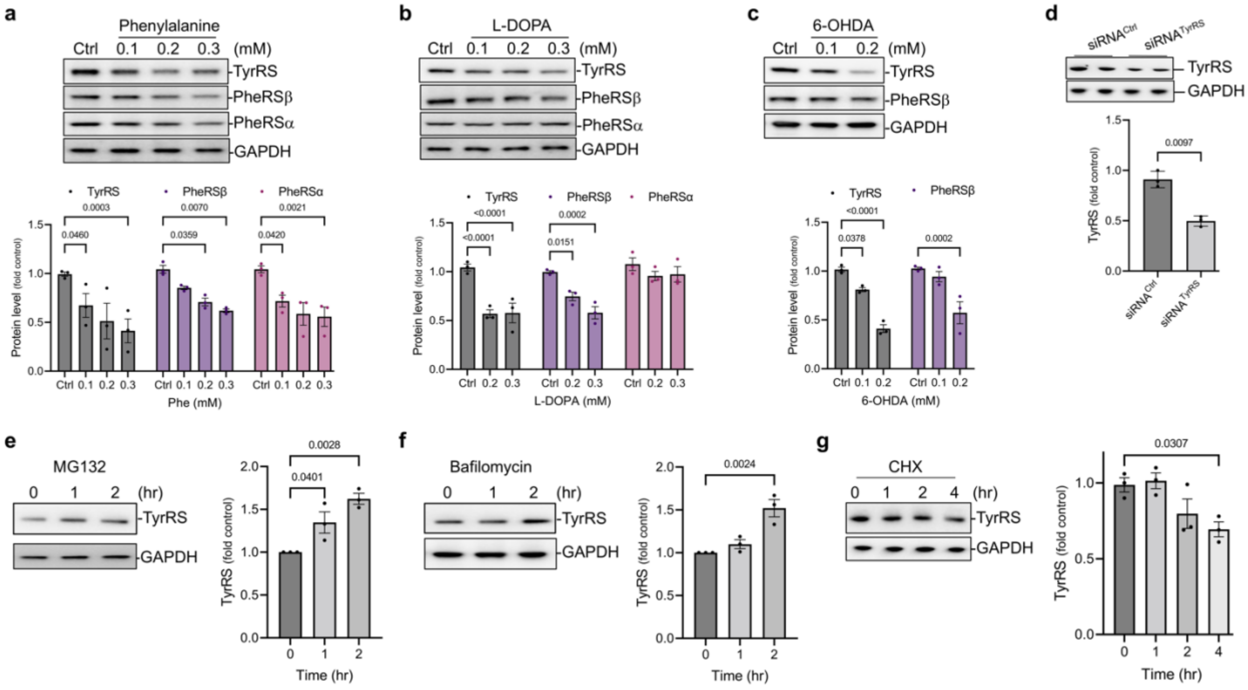

Supplementary Figure 3. Proteosome and lysosome facilitate the constitutive degradation of neuronal TyrRS. a. *Phenylalanine depletes TyrRS and PheRSα/β.* Primary cortical neurons were treated with Phe (100-300 μM) for 8 hr, and the levels of TyrRS, and PheRSα/β were detected by WB analysis using their specific antibodies. **b.** *L-DOPA decreases neuronal TyrRS.* Representative immunoblots showing TyrRS, PheRSα/β levels after treatment with increasing concentrations of L-DOPA (100-300 μM) for 8 hr using their specific antibodies. **c.** *6-OHDA decreases the levels of TyrRS.* Representative immunoblots showing TyrRS, PheRSβ levels after treatment with increasing concentrations of 6-OHDA (100 and 200 μM) for 8 hr using their specific antibodies. **d.** *TyrRS knockdown using siRNA in rat cortical neurons.* Rat cortical neurons (DIV 7) were transfected with siRNA against TyrRS or control siRNA (75 nM) for 72 hr, and the levels of TyrRS were quantified using anti-TyrRS antibody. **e, f**. *Inhibition of proteasome and autophagy increase the protein levels of TyrRS*. Primary cortical neurons were treated with MG132 (100 nM) (**e**) and bafilomycin (100 nM) (**f**) for upto 2 hr and the changes in the protein levels of TyrRS were immunoquantified using anti-TyrRS antibody. **g**. *Inhibition of protein synthesis decreases TyrRS protein levels*. Primary cortical neurons were treated with CHX (100 μg) for upto 4 hr and the changes in the protein levels of TyrRS were immunoquantified using anti-TyrRS antibody. All experiments in the figure were repeated 3 independent times and quantified for statistical significance.

Supplementary Figure 4

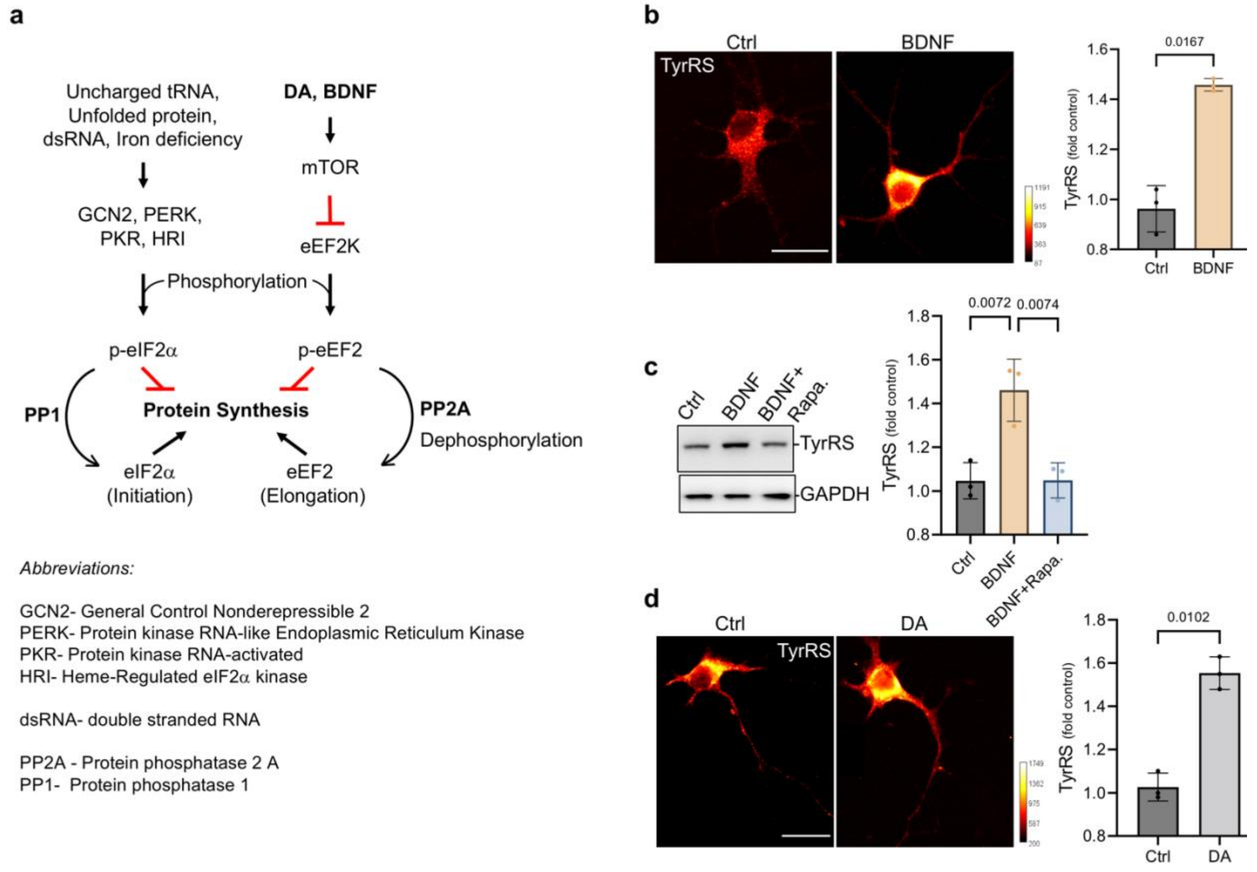

Supplementary Figure 4. BDNF stimulates the de novo synthesis of TyrRS in neurons. a. *Schematic representation of the mechanism of regulation of protein synthesis by the phosphorylation of eIF2α and eEF2*. **b, c**. *BDNF activates the de novo protein synthesis of TyrRS*. Representative images (scale bar, 10µm) for rat cortical neurons were treated with BDNF (50 nM) for 1hr either alone (**b**) or in combination with rapamycin (Rapa., 100 nM) (**c**), and subjected to either IF (**b**) or WB (**c**) analysis using anti-TyrRS antibody to detect changes in the protein levels of TyrRS. **d.** *DA increases neuronal TyrRS levels*. Representative images (scale bar, 10µm) for rat cortical neurons were treated with DA (200 µM) for 2 hr and subjected to IF analysis using anti-TyrRS antibody to detect changes in the protein levels of TyrRS. All experiments in the figure were repeated 3 independent times and quantified for statistical significance.

Supplementary Figure 5

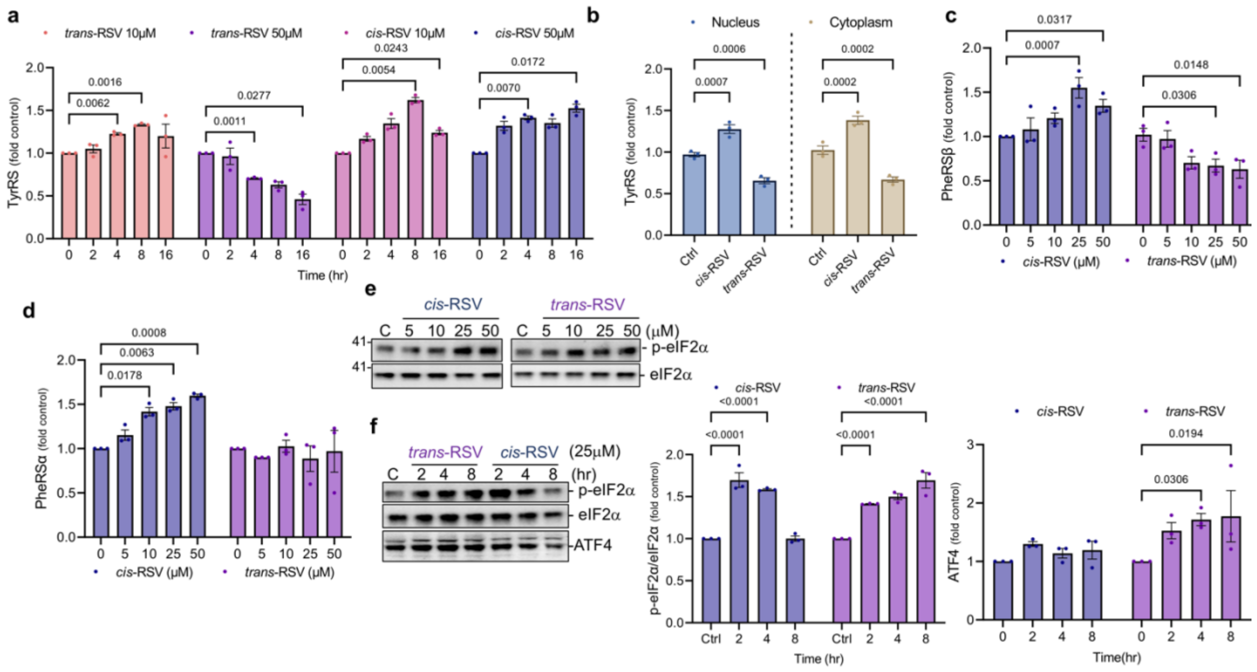

Supplementary Figure 5. *cis*-and *trans*-RSV have distinct effects on the phosphorylation status of eIF2α and eEF2. a-d. *Quantification of the WB and IF in Fig. 3a and b, and Fig. 3e.* **e.** *cis- and trans-RSV have differential effects on the phosphorylation of eIF2α.* Primary cortical neurons were treated with *cis*- and *trans*-RSV (5-50 μM)f for 2 hr and p-eIF2α were detected by WB analysis using anti-p-eIF2α antibody. **f.** *trans-RSV sustains the levels of p-eIF2α while the effect of cis-RSV is transient.* Primary cortical neurons were treated with *cis*- and *trans*-RSV (25 μM) for up to 8 hr and changes p-eIF2α were detected by WB analysis using anti-p-eIF2α antibody. The effect of p-eIF2α was also measured using the expression levels of ATF4. All experiments in the figure were repeated 3 independent times and quantified for statistical significance.

Supplementary Figure 6

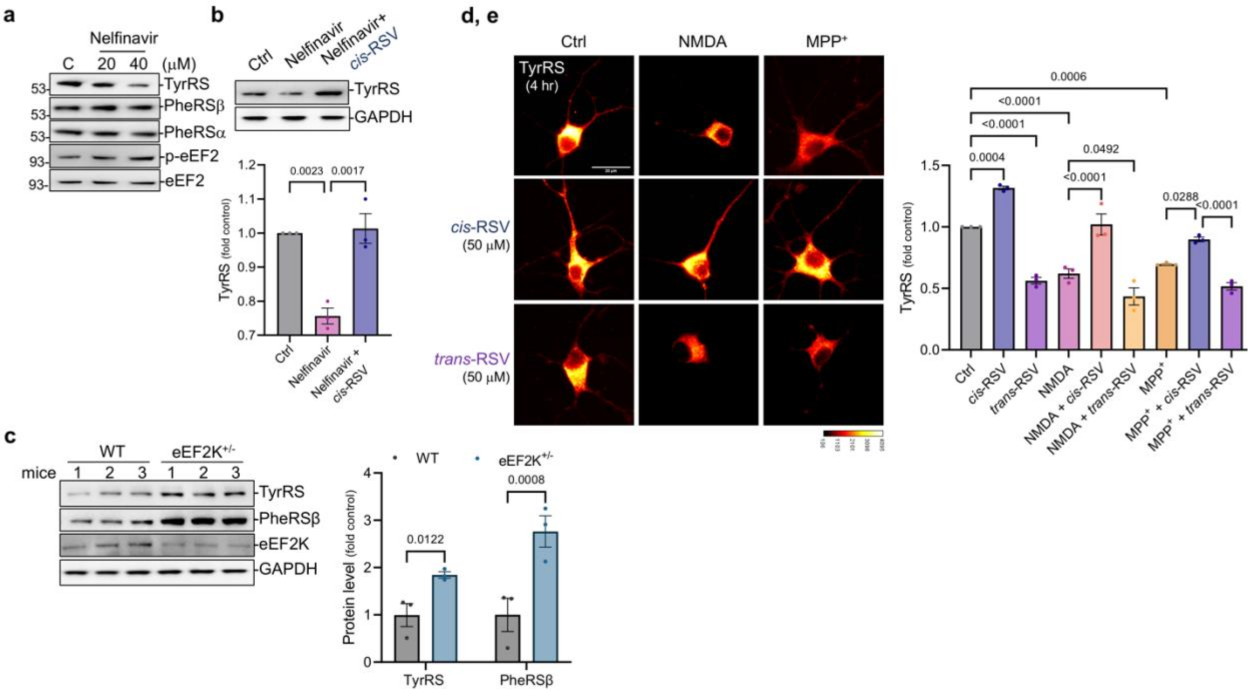

Supplementary Figure 6. Commonly used neurotoxic agents decrease TyrRS levels in rat cortical neurons. a. *Nelfinavir (eEF2K activator) depletes neuronal TyrRS.* Primary cortical neurons were treated with nelfinavir (20-40 µM) for 8 hr and changes in the levels of TyrRS, PheRSβ and p-eEF2 were detected by WB analysis using their corresponding antibodies. **b.** *cis-RSV protects neurons against nelfinavir-mediated depletion of TyrRS.* Primary cortical neurons were treated with nelfinavir (20 µM) alone or in combination with *cis*-RSV for 8 hr, and TyrRS was detected by WB analysis using anti-TyrRS antibody. **c.** *Genetic reduction of eEF2K increases TyrRS in vivo.* Quantitative western blots showing the protein levels of TyrRS and PheRSβ using anti-TyrRS and PheRSβ antibodies in the brain tissue samples from eEF2K$^{+/-}$ mice. **d**, **e**. *cis-RSV prevents, and trans-RSV exacerbates the decrease in the levels of TyrRS by neurotoxic agents.* Representative spectral images (scale bar, 20 µm) and quantification for neuronal TyrRS after treatment with neurotoxic agents (50 µM NMDA or 100 µM MPP^{+}) for 4 hr in combination with *cis-* and *trans*-RSV (50 µM) in rat cortical neurons (DIV 9). The graphical representation is for mean ± SEM TyrRS protein levels with statistical significance calculated using Student's paired t-test. All experiments in the figure were repeated 3 independent times and quantified for statistical significance.

Supplementary Figure 7

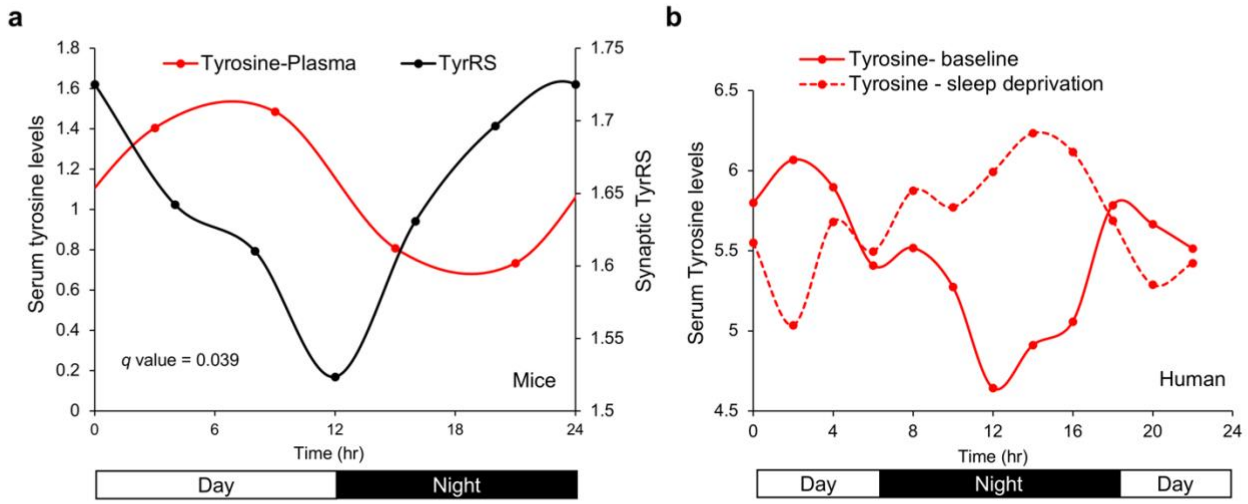

Supplementary Figure 7. Synaptic TyrRS is circadian-regulated, and sleep deprivation increases serum tyrosine levels in humans. a. *Synaptic TyrRS protein level is circadian-regulated.* Graph depicting the circadian protein levels of synaptic TyrRS and circadian levels of serum tyrosine were generated using data from the re-analysis of the mouse circadian proteomic (Noya, S. B. *et al.* (2019)) and metabolomic (Eckel-Mahan, K. L. *et al.* (2012)) data. **b**. *Human sleep deprivation disrupts circadian regulation of tyrosine.* Representative graph depicting the circadian levels of serum tyrosine of an individual was generated using data from the re-analysis of the human circadian metabolomic data (Grant, L. K. *et al.* (2019), and (Kervezee, L. *et al.* (2019)).

Supplementary Figure 8

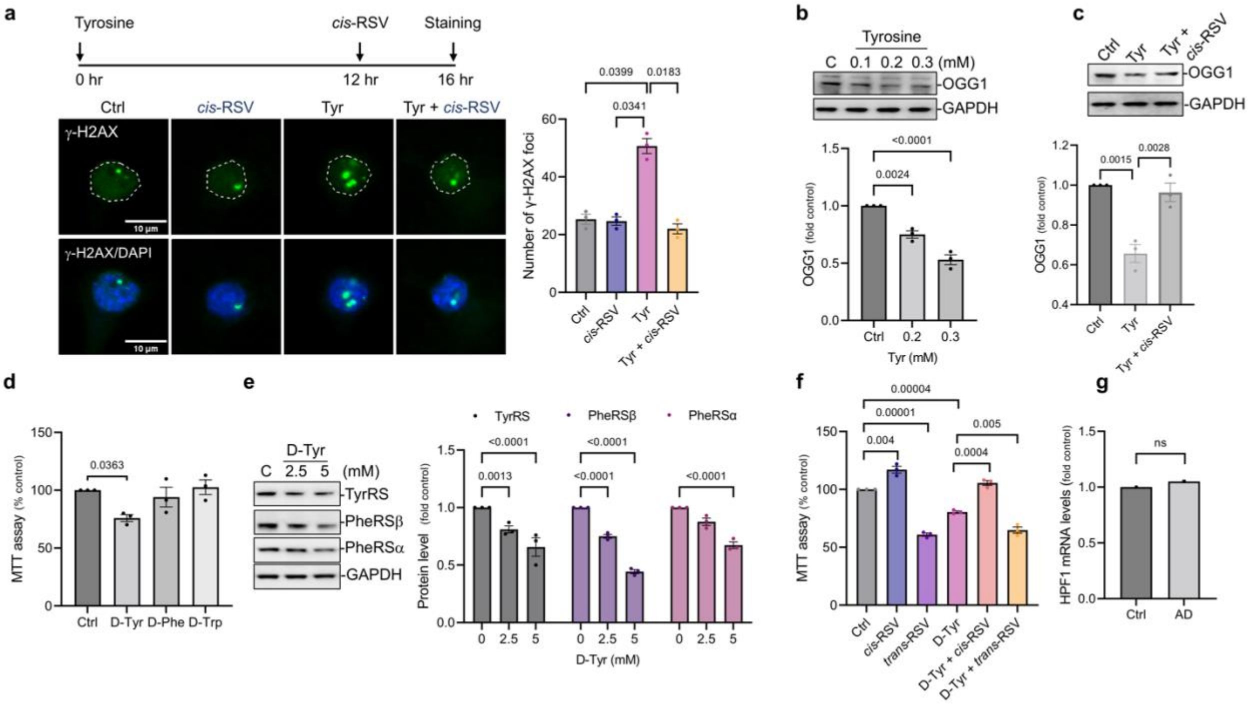

Supplementary Figure 8. D-tyrosine is neurotoxic and *cis*-RSV reverses existing neuronal DNA damage. a. *cis-RSV reverses existing neuronal DNA damage induced by tyrosine.* Immunostaining images (scale bar, 10 µm) for γ-H2AX (green; DAPI – nuclear marker, blue) in cortical neurons (DIV 9) after pre-treatment with Tyr (500 µM) for 12 hr, followed by the addition of *cis*-RSV (50 µM) for another 4 hr. **b**. *Tyrosine decreases the protein levels of OGG1*. Primary cortical neurons were treated with Tyr (100-300 µM) for 8 hr, and OGG1 was detected by WB analysis using anti-OGG1 antibody. **c**. *cis-RSV rescues tyrosine-mediated depletion of OGG1 protein.* Primary cortical neurons were treated with either Tyr (200 µM) alone or in combination with *cis*-RSV (50 µM) for 8 hr, and the levels of OGG1 was detected by WB analysis using anti-OGG1 antibody. **d**. *D-tyrosine induces neurotoxicity*. Rat cortical neurons (DIV 9) were treated with either D-Tyr or D-Phe or D-Trp (2 mM) for 48 hr, and viability was assessed using MTT assay. **e**. *D-Tyr decreases both TyrRS and PheRSβ proteins.* Primary cortical neurons were treated with D-Tyr (2.5 and 5 mM) for 8 hr, and the levels of TyrRS, and PheRSβ were detected by WB analysis using their specific antibodies. **f**. *cis-RSV protects neurons against D-tyrosine-induced toxicity and trans-RSV exacerbates it*. Rat cortical neurons (DIV 9) were treated with either D-Tyr alone or in combination with *cis* or *trans*-RSV (50 µM) for 48 hr, and viability was assessed using MTT assay. **g**. *mRNA levels of HPF1 are not affected in AD brains.* Graph depicting the HPF1 mRNA levels in the brains of AD patients using data from the re-analysis of the publicly available transcriptomics data as mentioned in Methods. All experiments in the figure were repeated 3 independent times and quantified for statistical significance.

Supplementary Figure 9

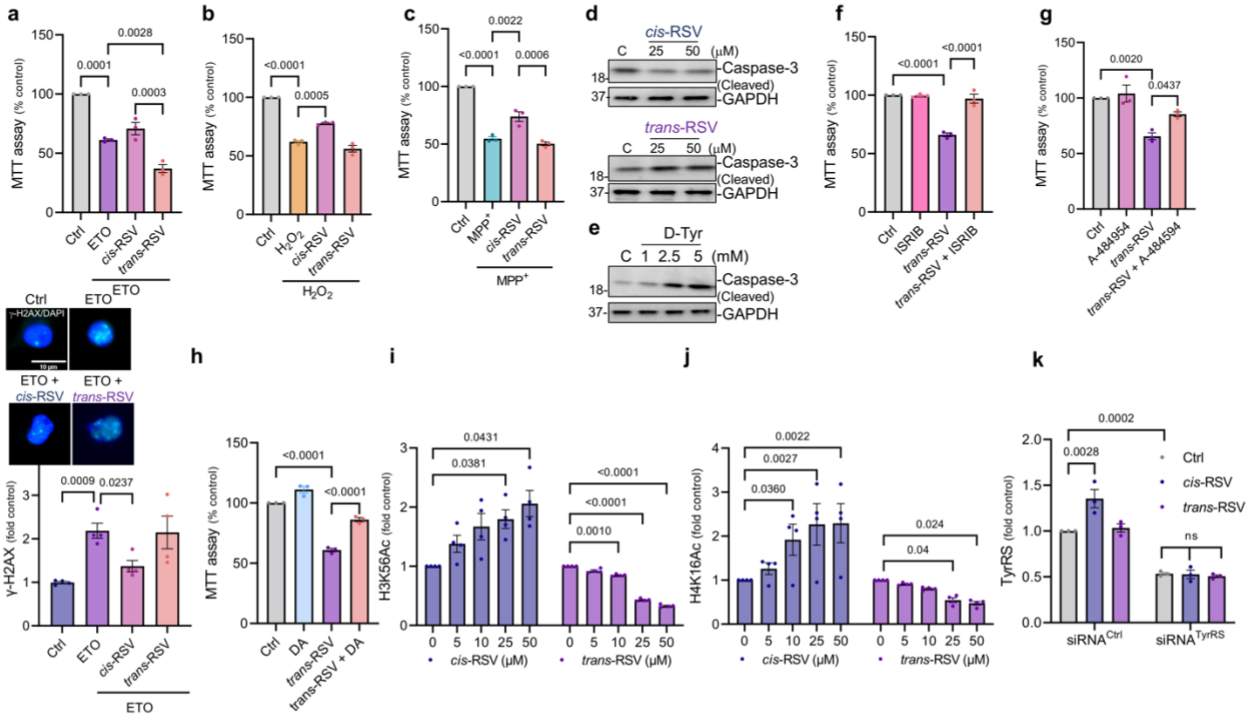

Supplementary Figure 9. Pharmacological activation of protein synthesis protects neurons against *trans*-RSV-mediated toxicity. a-**c**. *Only cis-RSV protects neurons against etoposide (**a**), H_2O_2 (**b**) and MPP^+(**c**) toxicity*. Rat cortical neurons (DIV 9) were exposed to 5 µM etoposide (ETO), 400 µM H_2O_2 or 10 µM MPP^+ for 24 hr after pre-treatment with *cis*-RSV or *trans*-RSV (50 µM) for 16 hr. Cell viability was assessed using MTT assay. (**a**) Immunostaining images (scale bar, 10 µm) for γ-H2AX foci (green; DAPI – nuclear marker, blue) in cortical neurons (DIV 10) after treatment with ETO (250 µM) alone or in combination with *cis*- and *trans*-RSV (50 µM) for 8 hr. **d**. *trans-RSV induces neuronal apoptosis*. Immunoblots showing the levels of cleaved caspase-3 in rat cortical neurons (DIV 9/10) after the treatment with *cis*- and *trans*-RSV (25 and 50 µM) for 24 hr. **e**. *D-tyrosine induces neuronal apoptosis*. Immunoblots showing the levels of cleaved caspase-3 in rat cortical neurons (DIV 9/10) after the treatment with D-Tyr (2 mM) for 16 hr. **f**. *ISRIB protects neurons from trans-RSV-mediated neurotoxicity*. Rat cortical neurons (DIV 8) were treated with *trans*-RSV alone or in combination with ISRIB (10 nM) for 48 hr and viability was measured using MTT assay. **g**. *A-484954 (eEF2K inhibitor) protects neurons from trans-RSV-mediated toxicity*. Rat cortical neurons (DIV 8) were treated with *trans*-RSV alone or in combination with A484954 (100 nM) for 48 hr and viability was measured using MTT assay. **h**. *DA protects neurons from trans-RSV-mediated toxicity*. Rat cortical neurons (DIV 8) were treated with *trans*-RSV alone or in combination with DA (100 µM) for 72 hr and viability was measured using MTT assay. **i** and **j**. *Quantification of immunoblot images for AcK16-H4 and AcK56-H3 levels respectively in Fig. 6e and f*. **k**. *Quantification of TyrRS protein levels in Fig. 6g*. All experiments in the figure were repeated 3 independent times and quantified for statistical significance.

Supplementary Figure 10

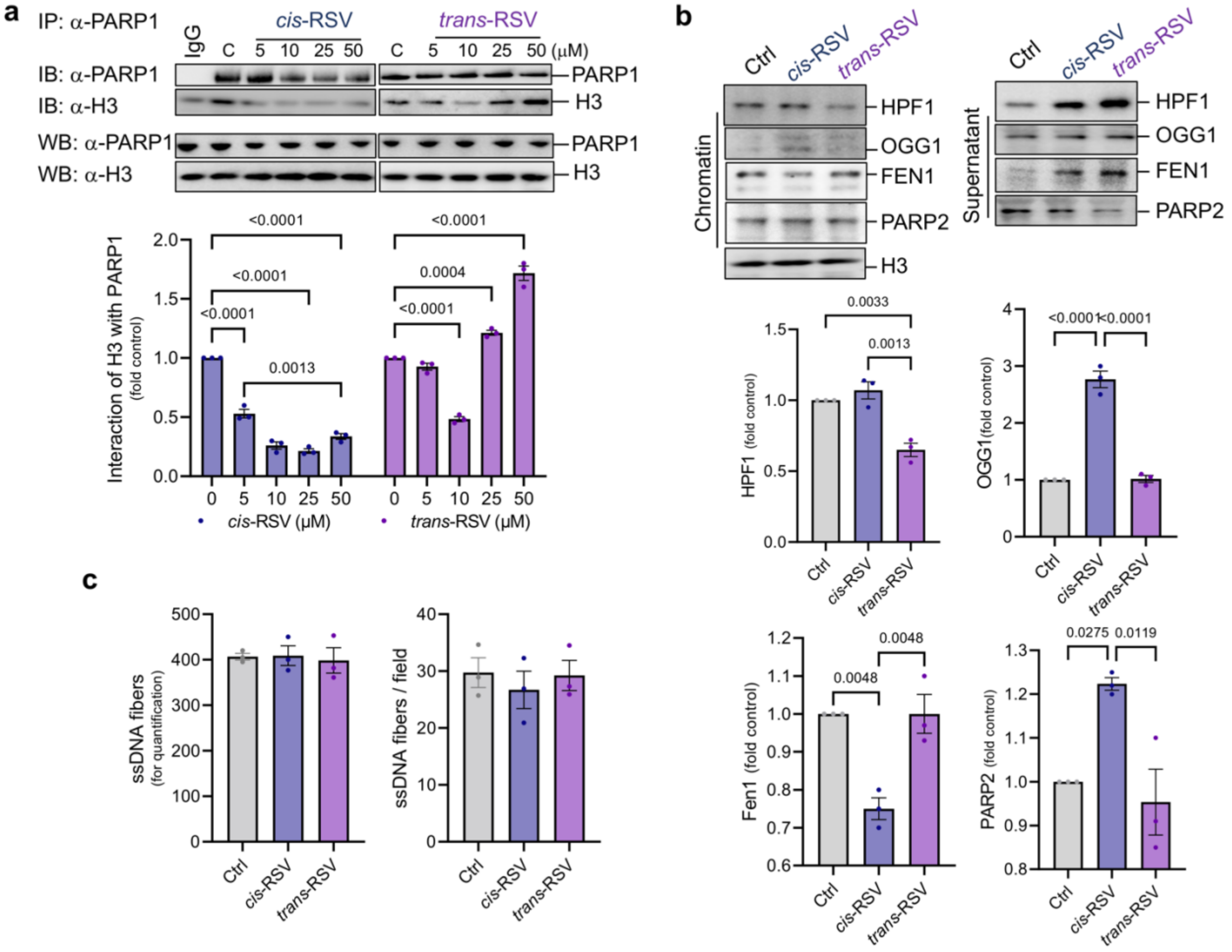

Supplementary Figure 10. *cis*-and *trans*-RSV have opposite effects on the recruitment of DNA repair factors to the chromatin. a. *cis-RSV prevents and trans-RSV increases the interaction of PARP1 with histone H3.* Representative immunoblots and quantification of the interaction of H3 with PARP1. Cortical neurons (DIV 9) were treated with *cis*- and *trans*-RSV (5-50 µM) for 30 min and PARP1 was immunoprecipitated (IP) using antibody against PARP1. The interaction of PARP1 with H3 was determined using anti-H3 antibody. **b**. *cis-RSV and trans-RSV have differential effects on chromatin recruitment of DNA repair proteins*. Representative immunoblots using specific antibodies, and quantification from chromatin fraction of cortical neurons (DIV 9) depicting HPF1, OGG1, and FEN1 after treatment with *cis*- and *trans*-RSV (50 µM) for 1 hr. **c**. *Quantification of neuronal DNA fibers for fig. 5d.* All experiments in the figure were repeated 3 independent times and quantified for statistical significance.

Supplementary Table 1

Patient demographics

Diagnosis	Sex	Age	Braak Stage	CERAD Score
No dementia	F	92	III	Sparse
No dementia	M	86	III	Absent
No dementia	F	95	III	Absent
No dementia	F	92	III	Sparse
No dementia	M	78	II	Sparse
No dementia	M	86	III	Absent
No dementia	F	91	III	Absent
AD	M	88	VI	Moderate
AD	M	91	V	Moderate
AD	F	96	VI	Frequent
AD	F	98	IV	Frequent
AD	M	88	VI	Moderate
AD	M	91	V	Moderate
AD	F	90	V	Frequent

M: Male; F: Female

Supplementary Table 2

Plasma Tyrosine concentration	Age (mean)	Reference
64.1 ± 15.1 µM<br>59.8 ± 16.8 µM	46.2 ± 6.4 (n = 174, M)<br>43.6 ± 4.8 (n = 1150, F)	Andraos, *et al.*, 2021[4]
61 (54-70) µM<br>52 (47-61) µM<br>63 (55-69) µM<br>59 (50-75) µM	26 ± 4 (n = 68, M)<br>25 ± 5 (n = 72, F)<br>71 ± 7 (n = 32, M)<br>72 ± 8 (n = 42, F)	Caballero, *et al.*, 1991[5]
66.48 ± 17.29 µM<br>78.4 ± 15.24 µM<br>72.59 ± 17.04 µM<br>81.7± 17.69 µM	62.71(55-74, n = 317, F)<br>69.71 (62-81, n = 317, F)<br>62.69 (55-74, n = 273, M)<br>69.69 (62-81, n = 273, M)	Chak, *et al.*, 2019 [6]

Supplementary Table 3

Condition/Disease	Elevated Amino Acids	References
Aging	**Tyr**, Leu, Ile, Val, Gly, Gln	Chak, C.M, *et al.* 2019[6]<br>Darst BF, *et al*. 2019[7]<br>Hwangbo, N. *et al.* 2021[8]<br>Andraos, *et al.* 2021[4]<br>van de Rest, O., *et al.* 2017[9]<br>Ravaglia, G. *et al.* 2002[10]<br>Kochhar, S. *et al.* 2006[11]<br>Kouchiwa, T. *et al.* 2012[12]<br>Rist, M. J. *et al.* 2017[13]
Menopause	**Tyr**, Val, Glu, Ile, Gly	Auro, K. *et al.* 2014[14]
Sleep Deprivation	**Tyr**, **Phe**, Trp, Ile, Thr, Pro, Lys	Grant, L. K. *et al.* 2019[15]<br>Gehrman, P. *et al.* 2018[16]
Delirium	**Tyr**, Trp<br>**Tyr**, **Phe**, Trp, Val, Ser	Pandharipande, P. P. *et al*. 2009[17]<br>Watne, L. O. *et al.* 2016[18]<br>Cuperlovic-Culf, M. et al. 2021[19]
Alzheimer's Disease (AD)<br>and<br>Mild Cognitive Impairment (MCI)	**Tyr**, **Phe**, Trp, Asp, Thr, Glu, Gln, Met, His, Pro, Val and Ala (elevated in brain of AD patients)<br>**Tyr**, **Phe** (plasma for MCI)	Storga, D., *et al.* 1996[20]<br>Fekkes, D. *et al.* 1998[21]<br>Kim, Y. H. *et al.* 2019[22]<br>Huo, Z. *et al.* 2020[23]<br>Ravaglia, G. *et al.* 2004[24]<br>Ravaglia, G. *et al.* 2002[10]
Parkinson's Disease (PD)	**Tyr**, **Phe**	Jimenez-Jimenez, F. J. *et al.* 2020[25]<br>Vascellari, S. *et al.* 2020[26]<br>Zhao, H. *et al.* 2018[27]<br>Wu, J.F. et al. 2016[28]<br>T. Hatano, et al. 2016[29]
Autism Spectrum Disorders (ASD)	**Tyr**, **Phe**, Glu, Asn, Ala, Lys, Arg, Cys, His, Met, Ser, Val	Skalny, A. V. *et al.* 2020[30]<br>Zou, M. Y. *et al.* 2020[31]<br>Aldred, S. *et al.* 2003[32]
Traumatic brain injury (TBI)	**Tyr**, **Phe**	Vuille-Dit-Bille, *et al.* 2012[33]
Tyrosinemia I	**Tyr**	Bendadi, F. *et al.* 2014[34]
Tyrosinemia II	**Tyr**	R. Huhn, H. *et al.* 1998[35]
Tyrosinemia III	**Tyr**	Ellaway, C. J. *et al.* 2001[36]
Phenylketonuria (PKU)	**Phe**	Blau, N., *et al.* 2010[37]
Diabetes,<br>Obesity, and Insulin Resistance	**Tyr**, **Phe**, Leu, Ile, Val	Felig, P., *et al.* 1969[38]<br>Newgard, C. B., et al. 2009[39]<br>Wang, T. J. *et al.* 2011[40]<br>R. Yang, *et al.* 2014[41]<br>P. Wurtz, et al. 2014[42]<br>Hellmuth et al, 2016 [43]
Cardiovascular Disease (CVD)	**Tyr**, **Phe**	Wurtz, P. *et al.* 2015[44]<br>P. Welsh, et al. 2018[45]

Hypertension	**Tyr**, **Phe**, Leu, Ile, Val	Altorf-van der Kuil, W. *et al*. 2013[46]<br>F. Teymoori, *et al.* 2018[47]
Myocardial Ischemia-Reperfusion injury	**Tyr, Phe**	A. Surendran, *et al.* 2019[48]
Heart failure	**Tyr**, **Phe** Met, His	Stryeck, S. *et al.* 2019[49]<br>Murashige, D. *et al.* 2020[50]<br>Hakuno, D., *et al.* 2015[51]<br>Cheng, M. L. et al. 2015[52]
Stroke	**Phe**, His	Vojinovic, D. *et al*. 2020[53]
Liver Diseases (Cirrhosis Hepatitis)	**Tyr**, **Phe**, Trp	S. J. O'Keefe, *et al.* 1981[54]<br>C. H. Dejong, *et al.* 2007[55]<br>M. Y. Morgan, *et al.* 1982[56]<br>A. J. McCullough, *et al.* 1981[57]
Auto-Immune Disorders	**Tyr**, **Phe**	D. Blackmore, L. Li, *et al*. 2020[58]
Sepsis	**Tyr**, **Phe**, Cys, Met, Ala, Asp, Glu, Pro	H. R. Freund, *et al.* 1978[59]<br>H. Freund, *et al.* 1979[60]

Supplementary Table 4

Composition for low-tyrosine medium

Component	Conc. (mM)
Glycine	0.4
L-Alanine	0.022
L-Arginine hydrochloride	0.398
L-Asparagine-H_2O	0.005
L-Cysteine	0.26
L-Histidine hydrochloride-H_2O	0.2
L-Isoleucine	0.801
L-Leucine	0.801
L-Lysine hydrochloride	0.797
L-Methionine	0.201
L-Phenylalanine	0.4
L-Proline	0.067
L-Serine	0.4
L-Threonine	0.798
L-Tryptophan	0.078
L-Tyrosine	*0.2*
L-Valine	0.803
Calcium Chloride ($CaCl_2$) (anhyd.)	1.8
Magnesium Chloride (anhydrous)	0.813
Potassium Chloride (KCl)	5.33
Sodium Bicarbonate ($NaHCO_3$)	26.19
Sodium Chloride (NaCl)	68.96
Sodium Phosphate monobasic (NaH_2PO_4-H_2O)	0.905
Zinc sulfate ($ZnSO_4$-$7H_2O$)	0.0006
D-Glucose (Dextrose)	25
HEPES	10.92
Sodium Pyruvate	0.227

References

1 Xu, J. *et al.* Regional protein expression in human Alzheimer's brain correlates with disease severity. *Commun Biol* **2**, 43, doi:10.1038/s42003-018-0254-9 (2019).

2 Johnson, E. C. B. *et al.* Large-scale proteomic analysis of Alzheimer's disease brain and cerebrospinal fluid reveals early changes in energy metabolism associated with microglia and astrocyte activation. *Nat Med* **26**, 769-780, doi:10.1038/s41591-020-0815-6 (2020).

3 Anisimova, A. S. *et al.* Multifaceted deregulation of gene expression and protein synthesis with age. *Proc Natl Acad Sci U S A* **117**, 15581-15590, doi:10.1073/pnas.2001788117 (2020).

4 Andraos, S. *et al.* Population epidemiology and concordance for plasma amino acids and precursors in 11-12-year-old children and their parents. *Sci Rep-Uk* **11**, doi:ARTN 361910.1038/s41598-020-80923-9 (2021).

5 Caballero, B., Gleason, R. E. & Wurtman, R. J. Plasma amino acid concentrations in healthy elderly men and women. *Am J Clin Nutr* **53**, 1249-1252, doi:10.1093/ajcn/53.5.1249 (1991).

6 Chak, C. M. *et al.* Ageing Investigation Using Two-Time-Point Metabolomics Data from KORA and CARLA Studies. *Metabolites* **9**, doi:ARTN 4410.3390/metabo9030044 (2019).

7 Darst, B. F., Koscik, R. L., Hogan, K. J., Johnson, S. C. & Engelman, C. D. Longitudinal plasma metabolomics of aging and sex. *Aging (Albany NY)* **11**, 1262-1282, doi:10.18632/aging.101837 (2019).

8 Hwangbo, N. *et al.* A metabolomic aging clock using human CSF. *J Gerontol A Biol Sci Med Sci*, doi:10.1093/gerona/glab212 (2021).

9 van de Rest, O., Bloemendaal, M., de Heus, R. & Aarts, E. Dose-Dependent Effects of Oral Tyrosine Administration on Plasma Tyrosine Levels and Cognition in Aging. *Nutrients* **9**, doi:10.3390/nu9121279 (2017).

10 Ravaglia, G. *et al.* Plasma amino acid concentrations in healthy and cognitively impaired oldest-old individuals: associations with anthropometric parameters of body composition and functional disability. *Br J Nutr* **88**, 563-572, doi:10.1079/BJN2002700 (2002).

11 Kochhar, S. *et al.* Probing gender-specific metabolism differences in humans by nuclear magnetic resonance-based metabonomics. *Anal Biochem* **352**, 274-281, doi:10.1016/j.ab.2006.02.033 (2006).

12 Kouchiwa, T. *et al.* Age-related changes in serum amino acids concentrations in healthy individuals. *Clin Chem Lab Med* **50**, 861-870, doi:10.1515/cclm-2011-0846 (2012).

13 Rist, M. J. *et al.* Metabolite patterns predicting sex and age in participants of the Karlsruhe Metabolomics and Nutrition (KarMeN) study. *Plos One* **12**, e0183228, doi:10.1371/journal.pone.0183228 (2017).

14 Auro, K. *et al.* A metabolic view on menopause and ageing. *Nat Commun* **5**, 4708, doi:10.1038/ncomms5708 (2014).

15 Grant, L. K. *et al.* Circadian and wake-dependent changes in human plasma polar metabolites during prolonged wakefulness: A preliminary analysis. *Sci Rep* **9**, 4428, doi:10.1038/s41598-019-40353-8 (2019).

16 Gehrman, P. *et al.* Altered diurnal states in insomnia reflect peripheral hyperarousal and metabolic desynchrony: a preliminary study. *Sleep* **41**, doi:10.1093/sleep/zsy043 (2018).

17 Pandharipande, P. P. *et al.* Plasma tryptophan and tyrosine levels are independent risk factors for delirium in critically ill patients. *Intens Care Med* **35**, 1886-1892, doi:10.1007/s00134-009-1573-6 (2009).
18 Watne, L. O. *et al.* Increased CSF levels of aromatic amino acids in hip fracture patients with delirium suggests higher monoaminergic activity. *Bmc Geriatr* **16**, 149, doi:10.1186/s12877-016-0324-0 (2016).
19 Cuperlovic-Culf, M. *et al.* Metabolomics and computational analysis of the role of monoamine oxidase activity in delirium and SARS-COV-2 infection. *Sci Rep* **11**, 10629, doi:10.1038/s41598-021-90243-1 (2021).
20 Storga, D., Vrecko, K., Birkmayer, J. G. D. & Reibnegger, G. Monoaminergic neurotransmitters, their precursors and metabolites in brains of Alzheimer patients. *Neuroscience Letters* **203**, 29-32, doi:Doi 10.1016/0304-3940(95)12256-7 (1996).
21 Fekkes, D. *et al.* Abnormal amino acid metabolism in patients with early stage Alzheimer dementia. *J Neural Transm (Vienna)* **105**, 287-294, doi:10.1007/s007020050058 (1998).
22 Kim, Y. H. *et al.* Metabolomic Analysis Identifies Alterations of Amino Acid Metabolome Signatures in the Postmortem Brain of Alzheimer's Disease. *Exp Neurobiol* **28**, 376-389, doi:10.5607/en.2019.28.3.376 (2019).
23 Huo, Z. *et al.* Brain and blood metabolome for Alzheimer's dementia: findings from a targeted metabolomics analysis. *Neurobiol Aging* **86**, 123-133, doi:10.1016/j.neurobiolaging.2019.10.014 (2020).
24 Ravaglia, G. *et al.* Plasma amino acid concentrations in patients with amnestic mild cognitive impairment or Alzheimer disease. *Am J Clin Nutr* **80**, 483-488, doi:10.1093/ajcn/80.2.483 (2004).
25 Jimenez-Jimenez, F. J., Alonso-Navarro, H., Garcia-Martin, E. & Agundez, J. A. G. Cerebrospinal and blood levels of amino acids as potential biomarkers for Parkinson's disease: review and meta-analysis. *Eur J Neurol* **27**, 2336-2347, doi:10.1111/ene.14470 (2020).
26 Vascellari, S. *et al.* Gut Microbiota and Metabolome Alterations Associated with Parkinson's Disease. *mSystems* **5**, doi:10.1128/mSystems.00561-20 (2020).
27 Zhao, H. *et al.* Potential biomarkers of Parkinson's disease revealed by plasma metabolic profiling. *J Chromatogr B Analyt Technol Biomed Life Sci* **1081-1082**, 101-108, doi:10.1016/j.jchromb.2018.01.025 (2018).
28 Wu, J. F. *et al.* NMR analysis of the CSF and plasma metabolome of rigorously matched amyotrophic lateral sclerosis, Parkinson's disease and control subjects. *Metabolomics* **12**, doi:ARTN 10110.1007/s11306-016-1041-6 (2016).
29 Hatano, T., Saiki, S., Okuzumi, A., Mohney, R. P. & Hattori, N. Identification of novel biomarkers for Parkinson's disease by metabolomic technologies. *J Neurol Neurosurg Psychiatry* **87**, 295-301, doi:10.1136/jnnp-2014-309676 (2016).
30 Skalny, A. V. *et al.* Serum amino acid spectrum in children with autism spectrum disorder (ASD). *Res Autism Spect Dis* **77**, doi:ARTN 10160510.1016/j.rasd.2020.101605 (2020).
31 Zou, M. Y. *et al.* Identification of Amino Acid Dysregulation as a Potential Biomarker for Autism Spectrum Disorder in China. *Neurotoxicity Research*, doi:10.1007/s12640-020-00242-9 (2020).

32 Aldred, S., Moore, K. M., Fitzgerald, M. & Waring, R. H. Plasma amino acid levels in children with autism and their families. *Journal of Autism and Developmental Disorders* **33**, 93-97, doi:Doi 10.1023/A:1022238706604 (2003).
33 Vuille-Dit-Bille, R. N., Ha-Huy, R. & Stover, J. F. Changes in plasma phenylalanine, isoleucine, leucine, and valine are associated with significant changes in intracranial pressure and jugular venous oxygen saturation in patients with severe traumatic brain injury. *Amino Acids* **43**, 1287-1296, doi:10.1007/s00726-011-1202-x (2012).
34 Bendadi, F. *et al.* Impaired cognitive functioning in patients with tyrosinemia type I receiving nitisinone. *J Pediatr* **164**, 398-401, doi:10.1016/j.jpeds.2013.10.001 (2014).
35 Huhn, R. *et al.* Novel and recurrent tyrosine aminotransferase gene mutations in tyrosinemia type II. *Human Genetics* **102**, 305-313, doi:DOI 10.1007/s004390050696 (1998).
36 Ellaway, C. J. *et al.* Outcome of tyrosinaemia type III. *Journal of Inherited Metabolic Disease* **24**, 824-832, doi:Doi 10.1023/A:1013936107064 (2001).
37 Blau, N., van Spronsen, F. J. & Levy, H. L. Phenylketonuria. *Lancet* **376**, 1417-1427, doi:Doi 10.1016/S0140-6736(10)60961-0 (2010).
38 Felig, P., Marliss, E. & Cahill, G. F. Plasma Amino Acid Levels and Insulin Secretion in Obesity. *New Engl J Med* **281**, 811-+, doi:Doi 10.1056/Nejm196910092811503 (1969).
39 Newgard, C. B. *et al.* A Branched-Chain Amino Acid-Related Metabolic Signature that Differentiates Obese and Lean Humans and Contributes to Insulin Resistance. *Cell Metab* **9**, 311-326, doi:10.1016/j.cmet.2009.02.002 (2009).
40 Wang, T. J. *et al.* Metabolite profiles and the risk of developing diabetes. *Nat Med* **17**, 448-453, doi:10.1038/nm.2307 (2011).
41 Yang, R. *et al.* Association of branched-chain amino acids with carotid intima-media thickness and coronary artery disease risk factors. *Plos One* **9**, e99598, doi:10.1371/journal.pone.0099598 (2014).
42 Wurtz, P. *et al.* Metabolic signatures of adiposity in young adults: Mendelian randomization analysis and effects of weight change. *PLoS Med* **11**, e1001765, doi:10.1371/journal.pmed.1001765 (2014).
43 Hellmuth, C. *et al.* Tyrosine Is Associated with Insulin Resistance in Longitudinal Metabolomic Profiling of Obese Children. *J Diabetes Res*, doi:Artn 210890910.1155/2016/2108909 (2016).
44 Wurtz, P. *et al.* Metabolite profiling and cardiovascular event risk: a prospective study of 3 population-based cohorts. *Circulation* **131**, 774-785, doi:10.1161/CIRCULATIONAHA.114.013116 (2015).
45 Welsh, P. *et al.* Circulating amino acids and the risk of macrovascular, microvascular and mortality outcomes in individuals with type 2 diabetes: results from the ADVANCE trial. *Diabetologia* **61**, 1581-1591, doi:10.1007/s00125-018-4619-x (2018).
46 Altorf-van der Kuil, W. *et al.* Dietary amino acids and the risk of hypertension in a Dutch older population: the Rotterdam Study. *Am J Clin Nutr* **97**, 403-410, doi:10.3945/ajcn.112.038737 (2013).
47 Teymoori, F., Asghari, G., Mirmiran, P. & Azizi, F. High dietary intake of aromatic amino acids increases risk of hypertension. *J Am Soc Hypertens* **12**, 25-33, doi:10.1016/j.jash.2017.11.004 (2018).
48 Surendran, A., Aliani, M. & Ravandi, A. Metabolomic characterization of myocardial ischemia-reperfusion injury in ST-segment elevation myocardial infarction patients

undergoing percutaneous coronary intervention. *Sci Rep* **9**, 11742, doi:10.1038/s41598-019-48227-9 (2019).
49 Stryeck, S. *et al.* Serum Concentrations of Citrate, Tyrosine, 2- and 3- Hydroxybutyrate are Associated with Increased 3-Month Mortality in Acute Heart Failure Patients. *Sci Rep* **9**, 6743, doi:10.1038/s41598-019-42937-w (2019).
50 Murashige, D. *et al.* Comprehensive quantification of fuel use by the failing and nonfailing human heart. *Science* **370**, 364-+, doi:10.1126/science.abc8861 (2020).
51 Hakuno, D., Hamba, Y., Toya, T. & Adachi, T. Plasma amino acid profiling identifies specific amino acid associations with cardiovascular function in patients with systolic heart failure. *Plos One* **10**, e0117325, doi:10.1371/journal.pone.0117325 (2015).
52 Cheng, M. L. *et al.* Metabolic disturbances identified in plasma are associated with outcomes in patients with heart failure: diagnostic and prognostic value of metabolomics. *J Am Coll Cardiol* **65**, 1509-1520, doi:10.1016/j.jacc.2015.02.018 (2015).
53 Vojinovic, D. *et al.* Association of circulating metabolites in plasma or serum and risk of stroke: Meta-analysis from seven prospective cohorts. *Neurology*, doi:10.1212/WNL.0000000000011236 (2020).
54 O'Keefe, S. J. *et al.* Increased plasma tyrosine concentrations in patients with cirrhosis and fulminant hepatic failure associated with increased plasma tyrosine flux and reduced hepatic oxidation capacity. *Gastroenterology* **81**, 1017-1024 (1981).
55 Dejong, C. H., van de Poll, M. C., Soeters, P. B., Jalan, R. & Olde Damink, S. W. Aromatic amino acid metabolism during liver failure. *J Nutr* **137**, 1579S-1585S; discussion 1597S-1598S, doi:10.1093/jn/137.6.1579S (2007).
56 Morgan, M. Y., Marshall, A. W., Milsom, J. P. & Sherlock, S. Plasma amino-acid patterns in liver disease. *Gut* **23**, 362-370, doi:10.1136/gut.23.5.362 (1982).
57 McCullough, A. J., Czaja, A. J., Jones, J. D. & Go, V. L. The nature and prognostic significance of serial amino acid determinations in severe chronic active liver disease. *Gastroenterology* **81**, 645-652 (1981).
58 Blackmore, D. *et al.* Metabolomic profile overlap in prototypical autoimmune humoral disease: a comparison of myasthenia gravis and rheumatoid arthritis. *Metabolomics* **16**, 10, doi:10.1007/s11306-019-1625-z (2020).
59 Freund, H. R., Ryan, J. A., Jr. & Fischer, J. E. Amino acid derangements in patients with sepsis: treatment with branched chain amino acid rich infusions. *Ann Surg* **188**, 423-430, doi:10.1097/00000658-197809000-00017 (1978).
60 Freund, H., Atamian, S., Holroyde, J. & Fischer, J. E. Plasma Amino-Acids as Predictors of the Severity and Outcome of Sepsis. *Annals of Surgery* **190**, 571-576, doi:Doi 10.1097/00000658-197911000-00003 (1979).
