# Supplementary material for: Cis- and trans-resveratrol have opposite effects on histone serine-ADP-ribosylation and tyrosine induced neurodegeneration
Source: Nat Commun. 2022 Jun 10;13:3244. doi: 10.1038/s41467-022-30785-8 (PMC9187644; doi:10.1038/s41467-022-30785-8)
Supplement: Supplementary file 2 — Reporting Summary [file 41467_2022_30785_MOESM2_ESM.pdf]

nature portfolio

Corresponding author(s): Sajish Mathew

Last updated by author(s): May 3, 2022

Reporting Summary

Nature Portfolio wishes to improve the reproducibility of the work that we publish. This form provides structure for consistency and transparency in reporting. For further information on Nature Portfolio policies, see our Editorial Policies and the Editorial Policy Checklist.

Statistics

For all statistical analyses, confirm that the following items are present in the figure legend, table legend, main text, or Methods section.

n/a	Confirmed	
☐	☒	The exact sample size (*n*) for each experimental group/condition, given as a discrete number and unit of measurement
☒	☐	A statement on whether measurements were taken from distinct samples or whether the same sample was measured repeatedly
☐	☒	The statistical test(s) used AND whether they are one- or two-sided<br>*Only common tests should be described solely by name; describe more complex techniques in the Methods section.*
☒	☐	A description of all covariates tested
☒	☐	A description of any assumptions or corrections, such as tests of normality and adjustment for multiple comparisons
☐	☒	A full description of the statistical parameters including central tendency (e.g. means) or other basic estimates (e.g. regression coefficient) AND variation (e.g. standard deviation) or associated estimates of uncertainty (e.g. confidence intervals)
☐	☒	For null hypothesis testing, the test statistic (e.g. *F*, *t*, *r*) with confidence intervals, effect sizes, degrees of freedom and *P* value noted<br>*Give P values as exact values whenever suitable.*
☒	☐	For Bayesian analysis, information on the choice of priors and Markov chain Monte Carlo settings
☒	☐	For hierarchical and complex designs, identification of the appropriate level for tests and full reporting of outcomes
☒	☐	Estimates of effect sizes (e.g. Cohen's *d*, Pearson's *r*), indicating how they were calculated

Our web collection on statistics for biologists contains articles on many of the points above.

Software and code

Policy information about availability of computer code

Data collection	SoftMax Pro 7.0.3 (for data collection from SpectraMax), Image Lab 9 (for data collection from western blot membranes), Las X (for data collection from LeicaDMI6000 epifluorescent microscope)
Data analysis	GraphPad Prism 9.3.1 for statistical analysis, ImageJ 1.53c for quantification of western blots and

For manuscripts utilizing custom algorithms or software that are central to the research but not yet described in published literature, software must be made available to editors and reviewers. We strongly encourage code deposition in a community repository (e.g. GitHub). See the Nature Portfolio guidelines for submitting code & software for further information.

Data

Policy information about availability of data

All manuscripts must include a data availability statement. This statement should provide the following information, where applicable:

- Accession codes, unique identifiers, or web links for publicly available datasets
- A description of any restrictions on data availability
- For clinical datasets or third party data, please ensure that the statement adheres to our policy

Raw data files for all figures, supplementary figures, tables and supplementary tables have been submitted to Nature Communications andd are also available from authors without any restrictions.

Field-specific reporting

Please select the one below that is the best fit for your research. If you are not sure, read the appropriate sections before making your selection.

☒ Life sciences ☐ Behavioural & social sciences ☐ Ecological, evolutionary & environmental sciences

For a reference copy of the document with all sections, see nature.com/documents/nr-reporting-summary-flat.pdf

Life sciences study design

All studies must disclose on these points even when the disclosure is negative.

Sample size	No sample determination was performed since no inferences were made from small sample numbers to larger populations. ALl experiments were repeated at least three times independently to ensure reproducibility, which is standard practice in the field.
Data exclusions	No data was excluded.
Replication	All studies were done using at least in triplicate and actual n values are indicated in the text for all experiments.
Randomization	No randomization was required due to the nature of the study with no involvement of human subjects or drug testing on other organisms.
Blinding	There was no blinding as this was not a randomized controlled trial. Samples and controls were treated and analyzed following the exact same procedure. The risk of bias is limited due to the nature of experiments performed, as there was no preconceived outcome for the study.

Behavioural & social sciences study design

All studies must disclose on these points even when the disclosure is negative.

Study description	*Briefly describe the study type including whether data are quantitative, qualitative, or mixed-methods (e.g. qualitative cross-sectional, quantitative experimental, mixed-methods case study).*
Research sample	*State the research sample (e.g. Harvard university undergraduates, villagers in rural India) and provide relevant demographic information (e.g. age, sex) and indicate whether the sample is representative. Provide a rationale for the study sample chosen. For studies involving existing datasets, please describe the dataset and source.*
Sampling strategy	*Describe the sampling procedure (e.g. random, snowball, stratified, convenience). Describe the statistical methods that were used to predetermine sample size OR if no sample-size calculation was performed, describe how sample sizes were chosen and provide a rationale for why these sample sizes are sufficient. For qualitative data, please indicate whether data saturation was considered, and what criteria were used to decide that no further sampling was needed.*
Data collection	*Provide details about the data collection procedure, including the instruments or devices used to record the data (e.g. pen and paper, computer, eye tracker, video or audio equipment) whether anyone was present besides the participant(s) and the researcher, and whether the researcher was blind to experimental condition and/or the study hypothesis during data collection.*
Timing	*Indicate the start and stop dates of data collection. If there is a gap between collection periods, state the dates for each sample cohort.*
Data exclusions	*If no data were excluded from the analyses, state so OR if data were excluded, provide the exact number of exclusions and the rationale behind them, indicating whether exclusion criteria were pre-established.*
Non-participation	*State how many participants dropped out/declined participation and the reason(s) given OR provide response rate OR state that no participants dropped out/declined participation.*
Randomization	*If participants were not allocated into experimental groups, state so OR describe how participants were allocated to groups, and if allocation was not random, describe how covariates were controlled.*

Ecological, evolutionary & environmental sciences study design

All studies must disclose on these points even when the disclosure is negative.

Study description	*Briefly describe the study. For quantitative data include treatment factors and interactions, design structure (e.g. factorial, nested, hierarchical), nature and number of experimental units and replicates.*
Research sample	*Describe the research sample (e.g. a group of tagged Passer domesticus, all Stenocereus thurberi within Organ Pipe Cactus National Monument), and provide a rationale for the sample choice. When relevant, describe the organism taxa, source, sex, age range and any manipulations. State what population the sample is meant to represent when applicable. For studies involving existing datasets,*

	describe the data and its source.
Sampling strategy	*Note the sampling procedure. Describe the statistical methods that were used to predetermine sample size OR if no sample-size calculation was performed, describe how sample sizes were chosen and provide a rationale for why these sample sizes are sufficient.*
Data collection	*Describe the data collection procedure, including who recorded the data and how.*
Timing and spatial scale	*Indicate the start and stop dates of data collection, noting the frequency and periodicity of sampling and providing a rationale for these choices. If there is a gap between collection periods, state the dates for each sample cohort. Specify the spatial scale from which the data are taken*
Data exclusions	*If no data were excluded from the analyses, state so OR if data were excluded, describe the exclusions and the rationale behind them, indicating whether exclusion criteria were pre-established.*
Reproducibility	*Describe the measures taken to verify the reproducibility of experimental findings. For each experiment, note whether any attempts to repeat the experiment failed OR state that all attempts to repeat the experiment were successful.*
Randomization	*Describe how samples/organisms/participants were allocated into groups. If allocation was not random, describe how covariates were controlled. If this is not relevant to your study, explain why.*
Blinding	*Describe the extent of blinding used during data acquisition and analysis. If blinding was not possible, describe why OR explain why blinding was not relevant to your study.*

Did the study involve field work? ☐ Yes ☒ No

Reporting for specific materials, systems and methods

We require information from authors about some types of materials, experimental systems and methods used in many studies. Here, indicate whether each material, system or method listed is relevant to your study. If you are not sure if a list item applies to your research, read the appropriate section before selecting a response.

Materials & experimental systems

n/a	Involved in the study
☐	☒ Antibodies
☒	☐ Eukaryotic cell lines
☒	☐ Palaeontology and archaeology
☐	☒ Animals and other organisms
☐	☒ Human research participants
☒	☐ Clinical data
☒	☐ Dual use research of concern

Methods

n/a	Involved in the study
☒	☐ ChIP-seq
☒	☐ Flow cytometry
☒	☐ MRI-based neuroimaging

Antibodies

Antibodies used

Acetyl-Histone H3 (Lys56)	Cell Signaling Technology	4243
Acetyl-Histone H3 (Lys9)	Cell Signaling Technology	9649
ARH3	Proteintech	16504-1-AP
eEF2	Cell Signaling Technology	2332
eIF2α	Cell Signaling Technology	5324
Fen1	Proteintech	14768-1-AP
GAPDH	Cell Signaling Technology	2118
H3	Proteintech	17168-1-AP
H3-S10-ADP-Ribose	Bio-RAD	HCA357
H4	Proteintech	16047-1-AP
HPF1	Novus Biologicals	NBP1-93973
OGG1	Proteintech	15125-1-AP
PARP1	Proteintech	66520-1-Ig
PARP2	Abcam	ab177529
PheRSα	Proteintech	18121-1-AP
PheRSβ	Proteintech	16341-1-AP
Phospho-eEF2 (Thr56)	Cell Signaling Technology	2331
Phospho-eIF2α (Ser51)	Cell Signaling Technology	3398
Poly (ADP-Ribose) Polymer	Abcam	ab14459
PP2A C	Cell Signaling Technology	2038
Puromycin	Millipore	MABE343
TyrRS	Abcam	ab50961
β-Tubulin	Cell Signaling Technology	2128
MAP2	Abcam	Ab5392

phospho-histone H2AX (Ser139)	Cell Signaling Technology	9178
TyrRS	Novus Biologicals	NBP1-32551
8-hydroxy-2'-deoxyguanosine	Abcam	ab48508
Goat anti-Chicken IgY (H+L) Secondary Antibody, Alexa Fluor™ 647	Invitrogen	A-21449
Goat anti-Mouse IgG (H+L) Highly Cross-Adsorbed Secondary Antibody, Alexa Fluor™ Plus 594	Invitrogen	A32742
F(ab')2-Goat anti-Rabbit IgG (H+L) Cross-Adsorbed Secondary Antibody, Alexa Fluor™ Plus 488	Invitrogen	A48282
Goat anti-Mouse IgG (H+L) Highly Cross-Adsorbed Secondary Antibody, Alexa Fluor™ Plus 555	Invitrogen	A21424
Goat anti-Rat IgG (H+L) Highly Cross-Adsorbed Secondary Antibody, Alexa Fluor™ Plus 488	Invitrogen	A48262
ssDNA, clone 16-19	Millipore	MAB3034
BrdU Clone B44	BD Biosciences	347580
Anti-rabbit IgG, HRP-linked Antibody	Cell Signaling Technology	7074
Anti-mouse IgG, HRP-linked Antibody	Cell Signaling Technology	7076
Rabbit (DA1E) mAb IgG XP® Isotype Control	Cell Signaling Technology	3900
ATF4 (D4B8)	Cell Signaling Technology	11815
Cleaved Caspase-3 (Asp175)	Cell Signaling Technology	9664

Validation

Acetyl-Histone H3 (Lys56): https://www.cellsignal.com/products/primary-antibodies/acetyl-histone-h3-lys56-antibody/4243
Acetyl-Histone H3 (Lys9): https://www.cellsignal.com/products/primary-antibodies/acetyl-histone-h3-lys9-c5b11-rabbit-mab/9649
ARH3: https://www.ptglab.com/products/ADPRHL2-Antibody-16504-1-AP.htm
eEF2: https://www.cellsignal.com/products/primary-antibodies/eef2-antibody/2332
eIF2α: https://www.cellsignal.com/products/primary-antibodies/eif2a-d7d3-xp-rabbit-mab/5324
Fen1: https://www.ptglab.com/products/FEN1-Antibody-14768-1-AP.htm
GAPDH: https://www.cellsignal.com/products/primary-antibodies/gapdh-14c10-rabbit-mab/2118
H3: https://www.ptglab.com/products/Histone-H3-Antibody-17168-1-AP.htm
H3-S10-ADP-Ribose: https://www.bio-rad-antibodies.com/monoclonal/protein-peptide-tag-h3-s10-adp-ribose-antibody-abd33644-hca357.html
H4: https://www.ptglab.com/products/HIST1H4E-Antibody-16047-1-AP.htm
HPF1: https://www.novusbio.com/products/hpf1-antibody_nbp1-93973
OGG1: https://www.ptglab.com/products/OGG1-Antibody-15125-1-AP.htm
PARP1: https://www.ptglab.com/products/PARP1-Antibody-13371-1-AP.htm
PARP2: https://www.abcam.com/ab177529.pdf
FARSA: https://www.ptglab.com/products/FARSA-Antibody-18121-1-AP.htm
FARSB: https://www.ptglab.com/products/FARSB-Antibody-16341-1-AP.htm
Phospho-eEF2 (Thr56): https://www.cellsignal.com/products/primary-antibodies/phospho-eef2-thr56-antibody/2331
Phospho-eIF2α (Ser51) (D9G8): https://www.cellsignal.com/products/primary-antibodies/phospho-eif2a-ser51-d9g8-xp-rabbit-mab/3398
Anti-Poly (ADP-Ribose) Polymer antibody [10H]: https://www.abcam.com/poly-adp-ribose-polymer-antibody-10h-ab14459.html
PP2A C Subunit Antibody: https://www.cellsignal.com/products/primary-antibodies/pp2a-c-subunit-antibody/2038
Anti-Puromycin Antibody, clone 12D10: https://www.emdmillipore.com/US/en/product/Anti-Puromycin-Antibody-clone-12D10,MM_NF-MABE343
Anti-Tyrosyl tRNA synthetase/TyrRS antibody [YAR5H08] : https://www.abcam.com/tyrosyl-trna-synthetasetyrrs-antibody-yar5h08-ab50961.html
β-Tubulin: https://www.cellsignal.com/products/primary-antibodies/b-tubulin-9f3-rabbit-mab/2128
Anti-MAP2 antibody: https://www.abcam.com/map2-antibody-ab5392.html
phospho-histone H2AX (Ser139): https://www.cellsignal.com/products/primary-antibodies/phospho-histone-h2a-x-ser139-20e3-rabbit-mab/9718
TyrRS: https://www.novusbio.com/products/yars-antibody_nbp1-32551
Anti-8-Hydroxy-2'-deoxyguanosine antibody [N45.1]: https://www.abcam.com/8-hydroxy-2-deoxyguanosine-antibody-n451-ab48508.html
Goat anti-Chicken IgY (H+L) Secondary Antibody, Alexa Fluor™ 647: https://www.thermofisher.com/antibody/product/Goat-anti-Chicken-IgY-H-L-Secondary-Antibody-Polyclonal/A-21449
Goat anti-Mouse IgG (H+L) Highly Cross-Adsorbed Secondary Antibody, Alexa Fluor™ Plus 594 : https://www.thermofisher.com/antibody/product/Goat-anti-Mouse-IgG-H-L-Highly-Cross-Adsorbed-Secondary-Antibody-Polyclonal/A32742
F(ab')2-Goat anti-Rabbit IgG (H+L) Cross-Adsorbed Secondary Antibody, Alexa Fluor™ Plus 488: https://www.thermofisher.com/antibody/product/Goat-anti-Rabbit-IgG-H-L-Cross-Adsorbed-Secondary-Antibody-Polyclonal/A48282
Goat anti-Mouse IgG (H+L) Highly Cross-Adsorbed Secondary Antibody, Alexa Fluor™ Plus 555 : https://www.thermofisher.com/antibody/product/Goat-anti-Mouse-IgG-H-L-Highly-Cross-Adsorbed-Secondary-Antibody-Polyclonal/A-21424
Goat anti-Rat IgG (H+L) Highly Cross-Adsorbed Secondary Antibody, Alexa Fluor™ Plus 488 : https://www.thermofisher.com/antibody/product/Goat-anti-Rat-IgG-H-L-Highly-Cross-Adsorbed-Secondary-Antibody-Polyclonal/A48262
ssDNA, clone 16-19: https://www.emdmillipore.com/US/en/product/Anti-DNA-Antibody-single-stranded-clone-16-19,MM_NF-MAB3034
BrdU (B44): https://www.bdbiosciences.com/content/bdb/paths/generate-tds-document.us.347580.pdf
Anti-rabbit IgG, HRP-linked Antibody: https://www.cellsignal.com/products/secondary-antibodies/anti-rabbit-igg-hrp-linked-antibody/7074

Anti-mouse IgG, HRP-linked Antibody: https://www.cellsignal.com/products/secondary-antibodies/anti-mouse-igg-hrp-linked-antibody/7076
Rabbit (DA1E) mAb IgG: https://www.cellsignal.com/products/primary-antibodies/rabbit-da1e-mab-igg-xp-isotype-control/3900
ATF4: https://www.cellsignal.com/products/primary-antibodies/atf-4-d4b8-rabbit-mab/11815
Cleaved Caspase-3 (Asp175) (5A1E): https://www.cellsignal.com/products/primary-antibodies/cleaved-caspase-3-asp175-5a1e-rabbit-mab/9664

Eukaryotic cell lines

Policy information about cell lines

Cell line source(s)	None used
Authentication	Not applicable
Mycoplasma contamination	Not applicable
Commonly misidentified lines (See ICLAC register)	Not applicable

Palaeontology and Archaeology

Specimen provenance	Not applicable
Specimen deposition	Not applicable
Dating methods	Not applicable

☐ Tick this box to confirm that the raw and calibrated dates are available in the paper or in Supplementary Information.

Ethics oversight	*Identify the organization(s) that approved or provided guidance on the study protocol, OR state that no ethical approval or guidance was required and explain why not.*

Note that full information on the approval of the study protocol must also be provided in the manuscript.

Animals and other organisms

Policy information about studies involving animals; ARRIVE guidelines recommended for reporting animal research

Laboratory animals	All mice were housed at the Wake Forest School of Medicine barrier facility under the supervision of the Animal Research Program. Mice adhered to a 12-hour light/12-hour dark cycle, with regular feeding, cage cleaning, and 24-hour food and water access. Both male and female mice were used for experimentation. All genotyping was determined by PCR. All animal samples were collected at

	age 8 to 9 months.
Wild animals	None used
Field-collected samples	None used
Ethics oversight	All protocols involving animals were approved by the Institutional Animal Care and Use Committee of Wake Forest University School of Medicine. Mice were kept in compliance with the NIH Guide for the Care and Use of Laboratory Animals (National Academies Press, 2011).

Note that full information on the approval of the study protocol must also be provided in the manuscript.

Human research participants

Policy information about studies involving human research participants

Population characteristics	All postmortem human tissue was obtained from the University of Washington School of Medicine Brain Bank. Diagnoses were based on cognitive testing, postmortem Braak staging (AD stages V–VI) stage, and Consortium to Establish a Registry for Alzheimer's Disease (CERAD) scores (59, 60). Studies were performed using hippocampal tissue from male and female patients clinically diagnosed with AD (n = 7) and age-matched controls (n = 7).
Recruitment	Not applicable
Ethics oversight	Samples of human tissue were collected in accordance with approved Institutional Review Board protocols. All patients gave informed consent.

Note that full information on the approval of the study protocol must also be provided in the manuscript.

Dual use research of concern

Policy information about dual use research of concern

Hazards

Could the accidental, deliberate or reckless misuse of agents or technologies generated in the work, or the application of information presented in the manuscript, pose a threat to:

No	Yes	
☒	☐	Public health
☒	☐	National security
☒	☐	Crops and/or livestock
☒	☐	Ecosystems
☒	☐	Any other significant area

Experiments of concern

Does the work involve any of these experiments of concern:

No	Yes	
☒	☐	Demonstrate how to render a vaccine ineffective
☒	☐	Confer resistance to therapeutically useful antibiotics or antiviral agents
☒	☐	Enhance the virulence of a pathogen or render a nonpathogen virulent
☒	☐	Increase transmissibility of a pathogen
☒	☐	Alter the host range of a pathogen
☒	☐	Enable evasion of diagnostic/detection modalities
☒	☐	Enable the weaponization of a biological agent or toxin
☒	☐	Any other potentially harmful combination of experiments and agents

ChIP-seq

Data deposition

☐ Confirm that both raw and final processed data have been deposited in a public database such as GEO.

☐ Confirm that you have deposited or provided access to graph files (e.g. BED files) for the called peaks.

Data access links<br>*May remain private before publication.*	*For "Initial submission" or "Revised version" documents, provide reviewer access links. For your "Final submission" document, provide a link to the deposited data.*
Files in database submission	*Provide a list of all files available in the database submission.*
Genome browser session<br>(e.g. UCSC)	*Provide a link to an anonymized genome browser session for "Initial submission" and "Revised version" documents only, to enable peer review. Write "no longer applicable" for "Final submission" documents.*

Methodology

Replicates	*Describe the experimental replicates, specifying number, type and replicate agreement.*
Sequencing depth	*Describe the sequencing depth for each experiment, providing the total number of reads, uniquely mapped reads, length of reads and whether they were paired- or single-end.*
Antibodies	*Describe the antibodies used for the ChIP-seq experiments; as applicable, provide supplier name, catalog number, clone name, and lot number.*
Peak calling parameters	*Specify the command line program and parameters used for read mapping and peak calling, including the ChIP, control and index files used.*
Data quality	*Describe the methods used to ensure data quality in full detail, including how many peaks are at FDR 5% and above 5-fold enrichment.*
Software	*Describe the software used to collect and analyze the ChIP-seq data. For custom code that has been deposited into a community repository, provide accession details.*

Flow Cytometry

Plots

Confirm that:

- [ ] The axis labels state the marker and fluorochrome used (e.g. CD4-FITC).
- [ ] The axis scales are clearly visible. Include numbers along axes only for bottom left plot of group (a 'group' is an analysis of identical markers).
- [ ] All plots are contour plots with outliers or pseudocolor plots.
- [ ] A numerical value for number of cells or percentage (with statistics) is provided.

Methodology

Sample preparation	*Describe the sample preparation, detailing the biological source of the cells and any tissue processing steps used.*
Instrument	*Identify the instrument used for data collection, specifying make and model number.*
Software	*Describe the software used to collect and analyze the flow cytometry data. For custom code that has been deposited into a community repository, provide accession details.*
Cell population abundance	*Describe the abundance of the relevant cell populations within post-sort fractions, providing details on the purity of the samples and how it was determined.*
Gating strategy	*Describe the gating strategy used for all relevant experiments, specifying the preliminary FSC/SSC gates of the starting cell population, indicating where boundaries between "positive" and "negative" staining cell populations are defined.*

- [ ] Tick this box to confirm that a figure exemplifying the gating strategy is provided in the Supplementary Information.

Magnetic resonance imaging

Experimental design

Design type	*Indicate task or resting state; event-related or block design.*
Design specifications	*Specify the number of blocks, trials or experimental units per session and/or subject, and specify the length of each trial or block (if trials are blocked) and interval between trials.*
Behavioral performance measures	*State number and/or type of variables recorded (e.g. correct button press, response time) and what statistics were used to establish that the subjects were performing the task as expected (e.g. mean, range, and/or standard deviation across subjects).*

Acquisition

Imaging type(s)	*Specify: functional, structural, diffusion, perfusion.*
Field strength	*Specify in Tesla*
Sequence & imaging parameters	*Specify the pulse sequence type (gradient echo, spin echo, etc.), imaging type (EPI, spiral, etc.), field of view, matrix size, slice thickness, orientation and TE/TR/flip angle.*
Area of acquisition	*State whether a whole brain scan was used OR define the area of acquisition, describing how the region was determined.*

Diffusion MRI ☐ Used ☐ Not used

Preprocessing

Preprocessing software	*Provide detail on software version and revision number and on specific parameters (model/functions, brain extraction, segmentation, smoothing kernel size, etc.).*
Normalization	*If data were normalized/standardized, describe the approach(es): specify linear or non-linear and define image types used for transformation OR indicate that data were not normalized and explain rationale for lack of normalization.*
Normalization template	*Describe the template used for normalization/transformation, specifying subject space or group standardized space (e.g. original Talairach, MNI305, ICBM152) OR indicate that the data were not normalized.*
Noise and artifact removal	*Describe your procedure(s) for artifact and structured noise removal, specifying motion parameters, tissue signals and physiological signals (heart rate, respiration).*
Volume censoring	*Define your software and/or method and criteria for volume censoring, and state the extent of such censoring.*

Statistical modeling & inference

Model type and settings	*Specify type (mass univariate, multivariate, RSA, predictive, etc.) and describe essential details of the model at the first and second levels (e.g. fixed, random or mixed effects; drift or auto-correlation).*
Effect(s) tested	*Define precise effect in terms of the task or stimulus conditions instead of psychological concepts and indicate whether ANOVA or factorial designs were used.*

Specify type of analysis: ☐ Whole brain ☐ ROI-based ☐ Both

Statistic type for inference (See Eklund et al. 2016)	*Specify voxel-wise or cluster-wise and report all relevant parameters for cluster-wise methods.*
Correction	*Describe the type of correction and how it is obtained for multiple comparisons (e.g. FWE, FDR, permutation or Monte Carlo).*

Models & analysis

n/a	Involved in the study
☐	☐ Functional and/or effective connectivity
☐	☐ Graph analysis
☐	☐ Multivariate modeling or predictive analysis

Functional and/or effective connectivity	*Report the measures of dependence used and the model details (e.g. Pearson correlation, partial correlation, mutual information).*
Graph analysis	*Report the dependent variable and connectivity measure, specifying weighted graph or binarized graph, subject- or group-level, and the global and/or node summaries used (e.g. clustering coefficient, efficiency, etc.).*
Multivariate modeling and predictive analysis	*Specify independent variables, features extraction and dimension reduction, model, training and evaluation metrics.*
